# Association between Malnutrition and Delirium in Older Chronic Kidney Disease Patients Admitted to Intensive Care Units: A Data Linkage Study

**DOI:** 10.1007/s12603-023-1938-5

**Published:** 2024-01-04

**Authors:** Ezinne O. Igwe, P. Ding, K.E. Charlton, J. Nealon, V. Traynor

**Affiliations:** 1 School of Medical, Indigenous and Health Sciences, Faculty of Science, Medicine and Health, University of Wollongong, 2522, Wollongong, NSW, Australia; 2 Illawarra Health and Medical Research Institute (IHMRI), Wollongong, NSW, Australia; 3 School of Mathematics and Applied Statistics, Faculty of Engineering and Information Sciences, University of Wollongong, Wollongong, New South Wales, Australia; 4 School of Nursing, Faculty of Science, Medicine and Health, University of Wollongong, Wollongong, New South Wales, Australia

**Keywords:** Delirium, older patients, malnutrition, chronic kidney disease, administrative data, intensive care unit

## Abstract

**Background:**

Independently, malnutrition and delirium in older hospitalised adults is prevalent. However, there is limited evidence on the association between these two conditions in older hospitalised adults with chronic kidney disease (CKD).

**Objectives:**

To determine the association between malnutrition and delirium in older CKD patients admitted to intensive care units (ICU).

**Methods:**

This data linkage study utilised administrative data from New South Wales (NSW) hospitals in Australia between 2017 and 2020. Admitted patient data was linked with Cause of Death Unit Record File, and NSW Registry of Deaths (RBD). The study population comprised all CKD patients aged 65 and over admitted to ICUs. Descriptive statistics were used to summarise patient characteristics. Binary logistic tested for association between malnutrition and delirium.

**Results:**

The study population included 748 CKD patients with a total 948 admissions in the study period. The International Statistical Classification of Diseases and Related Health Problems 10th Revision (ICD-10) was used to code for all outcomes and comorbidities. The incidence of delirium was 15.5% (n=141) and malnutrition was recorded in 11.3% (n=103) across all admissions. The adjusted odds ratio (OR) of a delirium diagnosis was 2.07 (95% CI: 1.27–3.39) for CKD patients that were malnourished compared to non-malnourished CKD patients.

**Conclusions:**

This study showed a significant association between delirium and malnutrition in older CKD patients admitted to ICU. Management of malnutrition could be critical in reducing the risk of delirium in older hospitalized patients with CKD. Additionally, more education and awareness around delirium and its association with malnutrition are needed in clinical practice.

## Abbreviations

APDC: Admitted Patient Data Collection
aOR: Adjusted Odds Ratio
CCI: Charlson Comorbidity Index
CKD: Chronic Kidney Disease
ICD: 10–International Classification of Diseases version 10
ICU: Intensive Care Unit
MDC: Major Diagnostic category
NSW: New South Wales (Australia).

## Introduction

With approximately 10% of the global adult population suffering from a form of CKD and associated mortality ranking in the top ten globally (1), malnutrition has been identified as an independent risk factor for morbidity and mortality in CKD patient populations (2). Ample evidence exists on the prevalence of malnutrition in patients living with CKD. This is attributed to malabsorption as a result of gut edema, poor appetite from cytokine production, difficulty in food preparation and consumption because of fatigue and difficulty breathing, as well as poor quality restrictive diets prescribed to reduce hyperkalaemia and hyperphosphatemia (3, 4). The global prevalence of malnutrition in CKD condition is estimated at 42.7% (range 35.2–50.6%) (5) however, there is limited evidence on malnutrition in CKD patients with an ICU admission. Patients admitted to the ICU are usually in a critical condition with reduced cognitive function and require specialised care which highlights a possible evidence gap in malnutrition in CKD patients. The association between CKD and different forms of cognitive dysfunction, including delirium and encephalopathy, is attributed to risk factors such as inflammation, uremic toxins, oxidative stress and an altered permeability of the blood brain barrier (6).

Delirium in the older CKD patient population is common and is characterised by an acute mental condition that is accompanied with an altered level of consciousness (7). A delirium diagnosis in hospital is linked to poor clinical outcomes including: 2–3 times higher risk of mortality; increased chances of relocation to a nursing home; as well as a heightened risk of developing and progressing dementia (8). Delirium prevalence in older patients admitted to ICU is estimated at up to 82% (9) with some of the risk factors for delirium including age older than 65 years, severe comorbidity, dehydration, and poor nutrition (9).

Although delirium and malnutrition in hospitalised older patients are independently well documented, there is limited evidence on the association between these two conditions in patients living with CKD and admitted to ICU. In different clinical contexts including in older orthopaedic surgery patients (10) and hospitalised older adults (11, 12), an association has been established between delirium and malnutrition with a paucity of evidence in CKD patient populations. Additionally, delirium and malnutrition are independently associated with CKD and this study could shed some light on the possible link between delirium and malnutrition in the older CKD patient population admitted in the ICU. This would subsequently strengthen the evidence on the association between these two conditions in different clinical populations.

Using administrative data, the aim of this study is to assess the association between delirium and malnutrition in CKD patients aged 65 and over and admitted to the ICU. Findings from this study could highlight gaps in the prevention and management of delirium in older hospitalised CKD patients, with a targeted focus on malnutrition during preoperative assessment.

## Methods

### Data source

This was a data linkage study using administrative data from New South Wales (NSW) hospitals in Australia. Hospital administrative data were collected between 2017 and 2020 from NSW Health Admitted Patient Data Collection (APDC) and is linked with Cause of Death Unit Record File, and NSW Registry of Deaths (RBD). The NSW Admitted Patient Data Collection (APDC) contains data for all hospital admissions from all public hospitals, public psychiatric hospitals, multipurpose services, private hospitals, and private day procedure centres in NSW. Approximately 400 facilities contribute the data held in the NSW APDC. Data included in the APDC include demographic information, diagnoses, procedures, and administrative information such as dates of admission and separation, source of referral to the service, service referred to on separation, and patient health insurance status.

The APDC data only records patient information for discharged individuals. Data on current hospital patients are not included. The linked datasets that were utilised were de-identified before analysis. This study was approved by the NSW Population and Health Services Research Ethics Committee.

### Data Linkage

The data linkage process was facilitated by the NSW Centre for Health Record Linkage (CHeReL) using ChoiceMaker software (13). The ChoiceMaker software utilises a probabilistic approach as well as an automated blocking algorithm and machine learning technique for ‘scoring' or assigning weights. ChoiceMaker also provides for standardisation and parsing and allows for the use of stacked data (multiple values available in a specific field) and includes a «transitivity engine» which allows for a user-specified action in the case of transitive linkage issues (14).

Prior to data linkage by NSW CHeReL, Health Information Managers or Clinical Coders in health information service departments in hospitals perform the disease and intervention coding (clinical coding). This is the process of assigning codes to diseases and interventions for each episode of admitted patient care in a public or private hospital within Australia. This process is facilitated by the use of ICD-10-AM and ACHI codes which are entered into computer systems (Patient Administration Systems) in public and private hospitals. Subsequently, these data are transmitted to the state health authority and to organisations such as the Commonwealth Department of Health and the Australian Institute of Health and Welfare.

### Study Population

The study population from the APDC dataset included all CKD patients aged 65 and over admitted to the ICU in New South Wales (NSW) hospitals over four years between 1 January 2017 and 31 December 2020. NSW is the largest state in Australia with a population of 8.166 million (15).

The study population was defined apriori to include CKD patients with an ICU admission and the following exclusion criteria were applied to the APDC dataset to create the final study population for the current study:

• Participants aged <65 or >111y at the time of admission (n=528,044) - this exclusion was necessary as evidence shows that the oldest individual in Australia is 111 years (16);
• A record without a CKD code as either the principal diagnosis or one of the 50 secondary diagnosis codes (n=6,740,381);
• Non-ICU hospital admissions (n=651,271).
• Records with negative length of stay (days) - this described LOS that were inaccurate. For example, there were LOS that were −12 days. This is expected as the use of administrative data has limitations, such as inaccurate data, coding errors, missing cases, and other inconsistencies; however, it is commonly the only source of information available to observe and analyse clinical issues (17).

**Figure 1:**
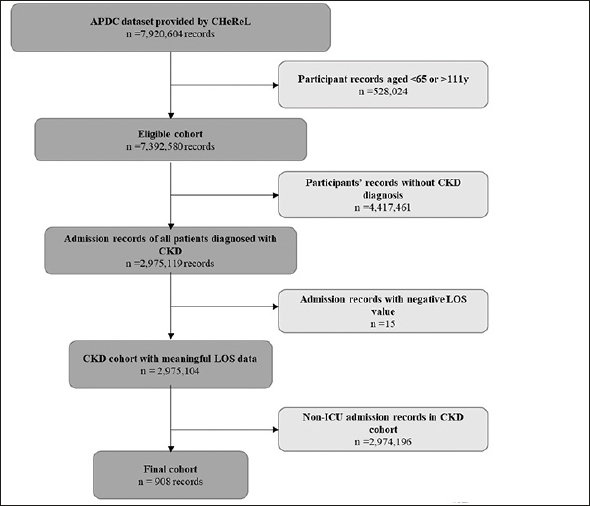
Eligible cohort and records included for analyses APDC: Admitted Patient Collection. CHeReL: Centre for Health Record Linkage. CKD: Chronic Kidney Disease ICU: Intensive Care Unit; LOS: length of stay

Following these exclusion criteria, the final study population had 908 admission records (from n=748 persons) (Figure 1). CKD events were identified by searching the ICD-10 diagnosis fields in each hospital record for CKD codes. All CKD events with an ICU admission were utilised for the current study. All malnutrition and delirium events were identified in a similar manner (see supplementary table 2 for ICD-10 codes for each condition). The order of ICU admission, delirium or malnutrition diagnoses could not be ascertained in this study.

### Statistical Analysis

Descriptive statistics were used to summarise participant characteristics. A binary logistic of generalized linear models was used to test for association between malnutrition and delirium incidence. Individual patients are fitted as random effect in the generalized linear mixed model to take account for the homogeneity of the observations occurred from the repeated admissions. The binary logistic model was adjusted for dementia diagnosis, Charlson comorbidity group (18), age group (categorical variable), and sex. The Charlson comorbidity index was categorised into three groups – severe comorbidity (CCI ≥ 3), mild-moderate comorbidity (CC1 = 1 or 2) and no reported comorbidity (CCI =0). There were seven categories for Major Diagnostic Category (MDC) (Table 1). MDCs usually correspond to an organ system or aetiology and are associated with a specific medical specialty (19). See Supplementary Table 1 for how MDCs were re-categorised into seven groups for the purpose of this analysis. Although, mortality data was linked to the admitted patient data, the cause of death in the study population was not described in the current study and no association between mortality and malnutrition or delirium was tested as this was outside of the scope of the current study aims. All analyses were performed using SAS 9.4 and IBM SPSS Statistics 27.

**Table 1:** Demographics of admissions according to nutritional status (n=908)

**Variable**	**All admissions (n=908)**	**Malnutrition at admission (n=103)**	**No malnutrition at admissions (n=805)**
Age, mean ± SD	77.1 ± 7.2	78.9 ± 7.2	76.8 ± 7.2
Age group, n (%)			
65 – 69 years	171 (18.8)	12 (11.7)	159 (19.8)
70 – 74 years	216 (23.8)	20 (19.4)	196 (24.3)
75 – 79 years	227 (25.0)	29 (28.2)	198 (24.6)
80 – 84 years	143 (15.7)	21 (20.4)	122 (15.2)
85+ years	151 (16.6)	21 (20.4)	130 (16.1)
Sex, n (%)			
Male	534 (58.8)	66 (64.1)	468 (58.1)
Female, n (%)	374 (41.2)	37 (35.9)	337 (41.9)
Charlson comorbidity group, n (%)			
‘Charlson=0'	91 (10.0)	3 (2.9)	88 (10.9)
‘Charlson=1,2'	290 (31.9)	30 (29.1)	260 (32.3)
‘Charlson>=3'	527 (58.0)	70 (68.0)	457 (56.8)
Comorbidities			
Dementia	15 (1.7)	5 (4.9)	10 (1.2)
Diabetes	620 (68.3)	63 (61.2)	557 (69.2)
Delirium diagnosis	141 (15.5)	29 (28.2)	112(13.9)
Length of stay, days	11.8 ± 16.3	22.3 ± 20.5	10.5 ± 15.3
Major Diagnostic category			
Brain, head and neck	90 (9.9)	7 (6.8)	83 (10.3)
CVD	372 (41.0)	31 (30.1)	341 (42.4)
Digestive system	139 (15.3)	14 (13.6)	125 (15.5)
Musculoskeletal, skin, and connective tissue	39 (4.3)	8 (7.8)	31 (3.9)
Kidney, urinary tract, and male reproductive system	77 (8.5)	10 (9.7)	67 (8.3)
Infection and injury and toxicity	123 (13.5)	19 (18.4)	104 (12.9)
Others	68 (7.5)	14 (13.6)	54 (6.7)
Local Health District			
*Major cities of Australia*			
Sydney LHD	50 (5.5)	12 (11.7)	38 (4.7)
S.Western Sydney LHD	107 (11.8)	13 (12.6)	94 (11.7)
S.Eastern Sydney LHD	29 (3.2)	7 (6.8)	22 (2.7)
W.Sydney LHD	144 (15.9)	7 (6.8)	137 (17.1)
N.Sydney LHD	33 (3.6)	3 (2.9)	30 (3.7)
Illawarra Shoalhaven LHD	89 (9.8)	15 (14.6)	74 (9.2)
Central Coast LHD	48 (5.3)	7 (6.8)	41 (5.1)
Hunter New England LHD	98 (10.8)	7 (6.8)	91 (11.4)
Northern NSW LHD	17 (1.9)	0 (0)	17 (2.1)
Mid North Coast LHD	17 (1.9)	0 (0)	17 (2.1)
*Inner/outer regional Australia*			
Southern NSW LHD	16 (1.8)	3 (2.9)	13 (1.6)
Murrumbidgee LHD	17 (1.9)	2 (1.9)	15 (1.9)
Western NSW LHD	59 (6.5)	7 (6.8)	52 (6.5)
Nepean Blue Mountains LHD	180 (19.8)	20 (19.4)	160 (20.0)
Recorded mortality, n (%)	384 (42.3)	64 (62.1)	320 (39.8)

## Results

### Descriptive Statistics

A total of 748 patients were included in this study population. There were 908 admissions in total. Malnutrition was recorded in 103 (11.34%) admissions. Table 1 describes the characteristics of the study population according to admissions. Since malnutrition and delirium are transient conditions, it is important to describe the study population by admission characteristics. There was no significant difference in the demographic characteristics of the study population by malnutrition status. The average age in this study population aged 65+y was 77.1 ± 7.2y (78.9 ± 7.2y for malnourished vs 76.8 ± 7.2y for non-malnourished category). Almost 60% of the population were male (58.5%) with a higher proportion of males in both malnutrition status groups (64.1% in malnourished vs 58.1% in non-malnourished groups). The proportion of participants with three or more comorbidities according to the Charlson comorbidity index was higher in the malnourished group (68%) compared to the non-malnourished group (56%). Mean length of stay in the malnourished group (22.3 ± 20.5 days) was twice that of the non-malnourished group (10.5 ± 15.3days). In addition, both dementia and delirium diagnosis were higher in the malnourished group compared to non-malnourished groups (28.2% vs 13.9 %, and 4.9% vs 1.2 %, respectively), as shown in Table 1. Results show that within the study period, 42% of the study population had died (62% in the malnourished group and 40% in the non-malnourished group).

### Association between malnutrition and delirium diagnosis in the study population

**Figure 2:**
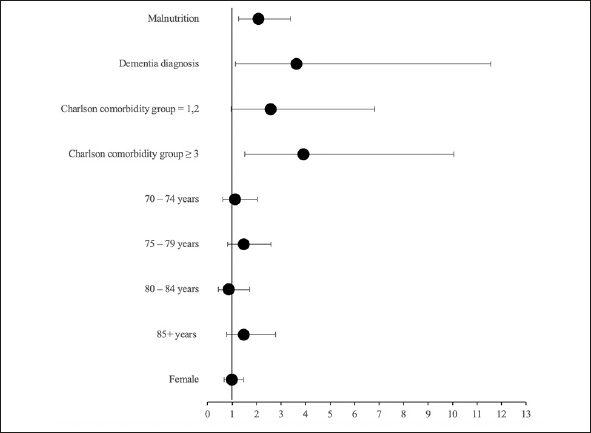
Adjusted odds ratio diagram of malnutrition as a risk factor for delirium diagnosis

Using a binary logistic of generalized linear model, which included malnutrition status and adjusted for dementia diagnosis, Charlson comorbidity group, age group, and sex, there was a significant association between delirium diagnosis and malnutrition (adjusted OR = 2.07 (95% CI: 1.27 – 3.39), p-value = 0.005. (Table 2 and Figure 2).

**Table 2:** Logistic regression analysis of potential risk factors for delirium diagnosis

**Variable**	**Adjusted Odds Ratio (95% CI)**	**p-value**
Nutritional status		
Non-malnourished	Reference	
Malnourished	2.07 (1.27–3.39)	0.005
Dementia diagnosis	3.62 (1.13 –11.56)	0.030
Charlson comorbidity group		
‘Charlson=0'	Reference	
‘Charlson=1,2'	2.58 (0.97 – 6.82)	0.056
‘Charlson>=3'	3.91 (1.52 – 10.05)	0.005
Age group		
65 – 69 years	Reference	
70 – 74 years	1.12 (0.62 – 2.03)	0.701
75 – 79 years	1.47 (0.82 – 2.59)	0.185
80 – 84 years	0.86 (0.43 – 1.72)	0.674
85+ years	1.47 (0.78 – 2.77)	0.230
Sex		
Male	Reference	
Female	0.997 (0.68 – 1.46)	0.99

## Discussion

This NSW data-linkage study on CKD patients aged 65 and over who had experienced an ICU admission during a 4-year study period found an independent association between delirium diagnosis and malnutrition, according to ICD-10 coding. Notably, the malnourished group had a two-fold likelihood of delirium diagnosis compared to the non-malnourished group (aOR = 2.07, 95% CI: 1.27–3.39). To the best of the authors' knowledge, this is a novel finding in a CKD population group, in this case, those admitted to the ICU.

Delirium has been documented as a common occurrence in patients admitted to the ICU with a cumulative incidence that exceeds 75% (20, 21), however, there is a paucity of evidence on the association between delirium and malnutrition in older patients within the acute care setting (11). Evidence suggest that in CKD patients, poor nutrition and the loss of water-soluble vitamins as a result of dialysis increases the risk of different forms of cognitive impairment (22). One study has suggested that with the brain's high nutritional requirement, malnutrition might play a significant role in the onset of cognitive dysfunction including delirium (11). Similar to our study results, a recent study found that in hospitalised, older, orthopaedic trauma patients, malnutrition increased the risk of developing delirium by two-fold (adjusted OR 2.07; 95% CI 1.01–1.35) (23). Another study observed similar results in patients with acute cardiovascular disease using three indices to assess nutritional status (i.e. Geriatric Nutritional Risk Index (GNRI), Prognostic Nutritional Index (PNI), and Controlling Nutritional Status (CONUT)) (20). On the other hand, ample evidence exists on the association between malnutrition and postoperative delirium (POD). POD is one of the most common surgical complication in older patient populations with incidence varying according to surgery type (24), from 15 to 25% after major elective surgery to 50% after high-risk procedures such as hip-fracture repair and cardiac surgery (24). One study explored whether poor nutritional status, as detected using the Mini Nutritional Assessment-Short Form (MNA-SF), is an indicator of developing POD in a population of older adults with hip fracture (25). Results showed that risk of malnutrition and overt malnutrition are independent predictors of postoperative delirium (25). In addition, a systematic review on the incidence and associated factors of delirium after orthopaedic surgery in older patients (≥ 65 years) showed a significant correlation between malnutrition and POD (10). The evidence between delirium and malnutrition in acute care settings has been consistent however, contrasting results have been reported for the association between malnutrition and POD. One study that investigated emergence delirium - following general anaesthetic, in older patients after non-cardiac surgery found that, although malnutrition was common in older patients undergoing non-cardiac surgery, there was no observed association with emergence delirium after adjusting for confounders (26). Although there are substantial differences in the current study population compared to all other reported studies (CKD patients with an admission to the ICU vs CVD, orthopaedic, and surgical patients), malnutrition may be a consistent indicator on the risk of delirium in hospitalised older patients.

Delirium is preventable in 30% to 40% of cases (27) and evidence from this study highlights the need for vigilance in terms of early detection of malnutrition in older patients as a preventive mechanism against the development of delirium. The proportion of the current study population diagnosed with delirium was 15.5% - a lower incidence compared to available literature which could be attributed to the underreporting of delirium (28). In clinical settings, underreporting of delirium limits optimisation of multidisciplinary interventions, patient and family education, as well as clinical management (29). Screening for risk factors, including malnutrition at admission may identify patients who are at a higher risk of delirium during hospitalisation. This could subsequently mitigate some of the poor clinical outcomes associated with delirium during hospitalisation – including increased hospital costs, longer hospital stay, relocation to a nursing home and other morbidities (30). One study found that in individuals undergoing hip fracture surgery, a comprehensive geriatric consultation during preoperative assessment significantly reduced delirium incidence and severity by more than 30% and 50% respectively (31). Another study on nurse-led interdisciplinary intervention program on older hip fracture patients observed reduced duration and severity of delirium (32). Generally, there is consistent evidence on the effectiveness of comprehensive multidisciplinary consultation at preoperative stage to help prevent delirium and the use of non-pharmacological interventions in the management of delirium (33).

The risk factors for delirium are well documented and different guidelines have been developed for the prevention and management of delirium in hospitalised older patients globally (34, 35, 36). Common risk factors of delirium include age 65 years and over, known cognitive impairment or dementia, previous history of delirium, severe comorbidity, and current hip fracture (34, 35, 36). In the available delirium guidelines, malnutrition assessment is consistently highlighted as a precipitating factor for delirium however, oftentimes, the availability of guidelines does not inform clinical practice (37). In addition, screening for malnutrition as a delirium risk is often overlooked in studies conducted on delirium in hospitalised older patients. In one comprehensive meta-analysis on the associated factors of delirium in older orthopaedic surgery patients, only four of 15 studies reported on malnutrition as a factor to consider in regards to delirium in this patient population (10). Evidence from that meta-analysis highlights the need for a larger focus on routine malnutrition screening in relation to delirium prevention and management, especially in older hospitalised patients.

A strength of this study is evident from the study design, namely the use of a large administrative dataset in NSW, Australia to explore for the first time, the association between malnutrition and delirium in older CKD patients admitted to the ICU. In addition, the relatively unique clinical profile of the current study – CKD patients with an admission to the ICU, enhances the consistent evidence base of the intersection of malnutrition and delirium. In contrast, the lack of timeline on delirium diagnosis is a limitation worth mentioning. The use of hospital administrative data means that it is unclear when delirium was diagnosed and what proportion of the participants were admitted with delirium and the proportion that developed delirium during hospitalisation.

Other limitations in this study can be seen in the proportion of malnutrition which was 11.3%. Even though the current study population was defined by very specific parameters, this figure cannot be considered to reflect current malnutrition prevalence in the general older patient population, which is estimated at between 50% and 80% (25). It is noteworthy that completeness and uniformity on comorbidities across different sources of morbidity data have shown that chronic conditions are not recorded consistently in hospital administrative datasets (38). Additionally, there is a possibility that a substantial gap exists in malnutrition between the general CKD population and CKD patients admitted in the ICU. One study on hospitalised COVID-19 patients showed a malnutrition prevalence of 42.1% with prevalence reaching 66.7% in patients admitted from the ICU (39). Another study found an 11% difference in prevalence of malnutrition from the same study population between hospital-collected data research study data (40). In addition, another study found that, using the ICD coding system, the overall delirium occurrence was underestimated by 30% (41). Validation studies on malnutrition coding using the ICD system found shortcomings and recommends updated malnutrition diagnosis codes globally (40, 42). As such, the ICD coding system may not reflect the current malnutrition diagnostic process that support clinical decision-making in practice. This is a potential limitation of using administrative data in population-based studies.

In conclusion, in line with current evidence, there is a significant association between malnutrition and delirium in this population of CKD patients aged 65y and older admitted to the ICU. There is a need for more focus on routine malnutrition screening in relation to delirium prevention and management, especially in older hospitalised patients. Further research could explore the incidence of delirium in other malnourished populations, including those with and without CKD in the community and hospitals. In addition, more education and awareness around delirium is needed as part of the clinical care team training to increase vigilance in clinical practice. Finally, an important implication from this study is the need for more vigilance in relation to malnutrition for people with CKD and recommendation for future studies to investigate further the mechanistic link between delirium and malnutrition. In CKD populations, there are numerous comorbidities recorded including malnutrition. The subsequent association of malnutrition with clinical outcomes such as delirium may be neglected in the care and management of CKD patients.

## Data sharing plan

Data described in the manuscript, code book, and analytic code are not publicly available and can be obtained from contacting the Centre for Health Record Linkage (CHeReL), NSW Ministry of Health.

## Electronic Supplementary Material

Association between Malnutrition and Delirium in Older Chronic Kidney Disease Patients Admitted to Intensive Care Units: A Data Linkage Study
