# Supplementary material for: Association between Malnutrition and Delirium in Older Chronic Kidney Disease Patients Admitted to Intensive Care Units: A Data Linkage Study
Source: J Nutr Health Aging. 2024 Jan 4;27(7):571–7. doi: 10.1007/s12603-023-1938-5 (PMC12929981; doi:10.1007/s12603-023-1938-5)
Supplement: Supplementary file 1 — Association between Malnutrition and Delirium in Older Chronic Kidney Disease Patients Admitted to Intensive Care Units: A Data Linkage Study [file mmc1.docx]

**Supplementary Table 2. ICD-10-AM codes for CKD, delirium, and malnutrition**

| **ICD-10 code** | **Description** |
| --- | --- |
| **CKD**  E08.2 | Diabetes mellitus due to underlying condition with kidney complications |
| E10.2 | Insulin-dependent diabetes mellitus with renal complications |
| E11.2 | Non-insulin-dependent diabetes mellitus with renal complications |
| E13.2 | Other specified diabetes mellitus with renal complications |
| E14.2 | Unspecified diabetes mellitus with renal complications |
| I12x | Hypertensive renal disease |
| I13x | Hypertensive heart and renal disease |
| N01x | Rapidly progressive nephritic syndrome |
| N02x | Recurrent and persistent haematuria |
| N03x | Chronic nephritic syndrome |
| N04x | Nephrotic syndrome |
| N06x | Isolated proteinuria with specified morphological lesion |
| N07x | Hereditary nephropathy, not elsewhere classified |
| N08x | Glomerular disorders in diseases classified elsewhere |
| N18x | Chronic kidney disease |
| N25x | Disorders resulting from impaired renal tubular function |
| Z49.0 | Preparatory care for dialysis |
| Z99.2 | Dependence on renal dialysis |
| **Delirium**  F05 | Delirium, not induced by alcohol and other psychoactive substances |
| **Malnutrition**  E40x -E46x | Malnutrition |
| R63.4 | Abnormal weight loss |
| R64 | Cachexia |
